# Diabetes mellitus as a risk factor for severe dengue fever and West Nile fever: A meta-analysis

**DOI:** 10.1371/journal.pntd.0012217

**Published:** 2024-05-31

**Authors:** Hong-Zheng Lu, Yu-Zhuang Xie, Chen Gao, Ying Wang, Ting-Ting Liu, Xing-Zhe Wu, Fang Dai, Duo-Quan Wang, Sheng-Qun Deng

**Affiliations:** 1 Department of Pathogen Biology, Anhui Province Key Laboratory of Zoonoses, the Key Laboratory of Zoonoses of High Institutions in Anhui, School of Basic Medical Sciences, Anhui Medical University, Hefei, China; 2 Department of Epidemiology and Biostatistics, School of Public Health, Anhui Medical University, Hefei, Anhui China; 3 Department of Tropical Medicine, College of Military Preventive Medicine, Army Medical University, Chongqing, China; 4 Department of Endocrinology, The First Affiliated Hospital of Anhui Medical University, Hefei, China; 5 National Institute of Parasitic Diseases, Chinese Center for Disease Control and Prevention (Chinese Center for Tropical Diseases Research), National Health Commission Key Laboratory of Parasite and Vector Biology; WHO Collaborating Center for Tropical Diseases; National Center for International Research on Tropical Diseases, Shanghai, China; University of California, Davis, UNITED STATES

## Abstract

**Background:**

Dengue fever (DF) and West Nile fever (WNF) have become endemic worldwide in the last two decades. Studies suggest that individuals with diabetes mellitus (DM) are at a higher risk of developing severe complications from these diseases. Identifying the factors associated with a severe clinical presentation is crucial, as prompt treatment is essential to prevent complications and fatalities. This article aims to summarize and assess the published evidence regarding the link between DM and the risk of severe clinical manifestations in cases of DF and WNF.

**Methodology/Principal findings:**

A systematic search was conducted using the PubMed and Web of Science databases. 27 studies (19 on DF, 8 on WNF) involving 342,873 laboratory-confirmed patients were included in the analysis. The analysis showed that a diagnosis of DM was associated with an increased risk for severe clinical presentations of both DF (OR 3.39; 95% CI: 2.46, 4.68) and WNF (OR 2.89; 95% CI: 1.89, 4.41). DM also significantly increased the risk of death from both diseases (DF: OR 1.95; 95% CI: 1.09, 3.52; WNF: OR 1.74; 95% CI: 1.40, 2.17).

**Conclusions/Significance:**

This study provides strong evidence supporting the association between DM and an increased risk of severe clinical manifestations in cases of DF and WNF. Diabetic individuals in DF or WNF endemic areas should be closely monitored when presenting with febrile symptoms due to their higher susceptibility to severe disease. Early detection and appropriate management strategies are crucial in reducing the morbidity and mortality rates associated with DF and WNF in diabetic patients. Tailored care and targeted public health interventions are needed to address this at-risk population. Further research is required to understand the underlying mechanisms and develop effective preventive and therapeutic approaches.

## Author summary

In our study, we investigated the association between diabetes mellitus (DM) and the risk of severe clinical manifestations in cases of dengue fever (DF) and West Nile fever (WNF). By analyzing 27 studies involving over 342,000 laboratory-confirmed patients, we found compelling evidence supporting a link between DM and an increased risk of severe complications in both DF and WNF. Moreover, DM was found to significantly raise the risk of mortality from these diseases.

Our findings emphasize the importance of early detection and appropriate management strategies for diabetic individuals residing in endemic areas. Healthcare providers should be vigilant in monitoring diabetic patients with febrile symptoms, as they are more susceptible to developing severe disease. Tailored care and targeted interventions are crucial to minimize the morbidity and mortality rates associated with DF and WNF in diabetic individuals.

These findings have significant implications for public health, highlighting the need for awareness campaigns and preventive measures aimed at diabetic individuals. Further research is needed to understand the underlying mechanisms and develop effective strategies for prevention and treatment. By addressing these issues, we can reduce the impact of DF and WNF on individuals with DM.

## Introduction

*Flaviviridae* are a family of small enveloped viruses comprising four genera: *Flavivirus*, *Pegivirus*, *Pestivirus*, and *Hepativirus*, which are host-specific and pathogenic, mostly infecting mammals and birds. Clinical signs of flaviviral infection range from asymptomatic to severe or fatal hemorrhagic fever or neurologic disease [1]. Many flaviviruses are transmitted through the bite of infected arthropod vectors, primarily the *Aedes* genus and *Culex* genus. Human-to-human transmission from infected blood and tissues is also possible [2]. Flaviviruses have caused serious public health problems over the past decades, with epidemics of dengue virus (DENV), Japanese encephalitis virus (JEV), West Nile virus (WNV), Zika virus (ZIKV), and yellow fever virus (YFV) occurring worldwide [3]. A study of DENV prevalence estimated that 3.9 billion people worldwide are at risk of DENV infection. Over the past 20 years, the number of dengue cases reported to the World Health Organization (WHO) has increased more than sevenfold, from 505,430 in 2000 to more than 2.4 million in 2010 and 5.2 million in 2019 [4,5]. WNV first appeared in the northeastern United States in 1999 and is now distributed throughout most of the United States and southern Canada [6].

Although most people infected with DENV and WNV present asymptomatically or with undifferentiated febrile illnesses, a small number of infected individuals develop acute fever that may progress to severe clinical manifestations such as hemorrhage, vascular leakage, and encephalitis [7]. Because dengue fever (DF) and West Nile fever (WNF) are characterized by dynamic clinical changes over time, it is of great practical importance to identify predictive factors that measure the evolution of the disease into severe illness in the early clinical stages [8,9]. Available evidence suggests that age, sex, genetic background, and comorbidities may adversely affect the clinical presentation of the infection [9–11]. However, current knowledge of the risk factors for both diseases is insufficient to predict whether a patient will develop more severe clinical symptoms or even die. Early indicators of dengue progression to a severe stage (including abdominal pain or pressure, bleeding from mucous membranes, liver enlargement of more than 2 centimeters, and erythrocyte pressurization accompanied by a rapid drop in platelet count) are described in the 2009 WHO dengue guidelines [12]. In areas where the disease has been prevalent for a long time, the monitoring cost of these indicators is high, and unnecessary medical resources may be wasted. In addition, some of the warning signs may appear after the disease has progressed, lacking sensitivity and clinical value [13]. For WNF, there is also a lack of a clinical presentation that has been shown to be specific enough to predict severe illness, and it is now generally accepted that preexisting chronic conditions such as obesity, asthma, diabetes mellitus (DM), and hypertension are risk predictors of severe illness in WNF [14,15]. Movement disorders (muscle spasms, Parkinson’s syndrome) in patients and multifocal chorioretinitis have also been reported to be predictive of the development of WNF [16,17].

When infection occurs, elucidating the factors influencing disease severity is critical to identifying populations at high risk for severe illness, and effective intervention programmes and individualized clinical surveillance practices should specifically target these populations. DM is a multifaceted disease involving chronic metabolic disorders and immune dysfunction that leads to a wide range of clinical complications [18,19]. Additionally, DM is one of the most common and efficient predictors of potential clinical deterioration of flaviviruses [20,21]. The purpose of this study was to systematically review the available literature on the course of DF and WNF diseases associated with DM, to further determine whether DM promotes flavivirus infections (DENV and WNV) and to assess the magnitude of its role in serious versus nonserious clinical outcomes of disease infections.

## Methods

### Literature search

For this systematic review and meta-analysis, we followed the protocol described in the PRISMA statement [22–24]. We searched two databases (PubMed, Web of Science) to access all relevant published articles as of August 1, 2023 describing the association between DM and DENV and WNV. We used the following keywords: (("diabetes") OR ("mellitus") OR ("glycuresis") OR ("alloxan diabetes") OR ("alloxandiabetes") OR ("maturity-onset diabetes")) AND (("mosquito") OR ("mosquito-borne disease") OR ("MBD") OR ("dengue") OR ("breakbone fever") OR ("DENV") OR ("classical dengues") OR ("West Nile")). Only publications in English were included in this study. Conference abstracts were not included due to the lack of detailed descriptions of the study methods, and thus, the subsequent quality assessment could not be performed.

### Inclusion and exclusion criteria

Two investigators (HZ Lu and YZ Xie) reviewed the titles and abstracts independently to identify the potentially eligible studies, for which the full texts were retrieved, and further assessment by reviewing the full text was conducted to identify the eligible studies. All discrepancies were resolved by discussion with the third investigator (C Gao).

The inclusion criteria of the studies in this meta-analysis were as follows: (1) DENV and WNV were clearly defined in the text; (2) both experimental and control groups were patients; and (3) reliable case identification methods were available.

The exclusion criteria were as follows: (1) no available full text or no extracted data; (2) fewer than 3 cases in each study or animal/cell study; (3) data from before 2000; and [4] when there were multiple publications on the same population or based on overlapping data, the latest or the largest study was included.

### Data extraction

The following data were independently extracted from the studies by two investigators (Y Wang and TT Liu): (1) information about the publication (article title, first author, year of publication, year of data, region, study design); (2) data acquisition methods and case identification methods; (3) number of participants in the experiment; and (4) criteria for case definition.

### Case definitions

#### Dengue

Combining the 1997 and 2009 WHO guidelines for the classification of critical illnesses, we classified dengue progression into two groups [5,12]:

(1) Based on routine clinical data collected by the WHO’s 1997 guidelines, these guidelines classify symptomatic dengue virus infections into three clinical categories: undifferentiated fever, DF, and denguehemorrhagic fever (DHF). DHF was further categorized into four severity levels, of which levels III and IV were defined as dengue shock syndrome (DSS). We refer to DHF/DSS as “severe clinical presentation of dengue”.

(2) Applying the WHO 2009 classification criteria for dengue, a severe dengue case is defined as a suspected dengue patient with one or more of the following diseases: (i) severe plasma leakage that leads to shock (dengue shock) and/or fluid accumulation with respiratory distress; (ii) severe bleeding; and (iii) severe organ impairment.

### West Nile fever

We refer to those who developed West Nile Neuroinvasive Disease (WNND) as having a “severe clinical presentation of WNV”, such as cases of WN encephalitis (WNE), WN meningitis (WNM), poliomyelitis or acute flaccid paralysis, WNV-associated retinopathy (WNVR), chorioretinitis or fatal cases [15,25–31].

### Quality assessment

The qualities of the included studies were assessed by the Newcastle Ottawa Quality Scale (NOS) [32]. This scale evaluates the quality of the study by 8 questions from three aspects, namely, adequate case definition; representativeness of the cases; selection of controls; definition of controls; comparability of cases and controls on the basis of the design or analysis; ascertainment of exposure; same method of ascertainment for cases and controls; and nonresponse rate. For each trial, the results of the assessment were given. The quality assessment was performed by two investigators independently (LHZ and GC), and discrepancies were resolved by discussion with the third investigator (DSQ).

### Statistical analysis

Meta-analyses were performed using Stata 16.0. We used odds ratios as the “primary model” and used random-effects or fixed-effects meta-analysis across all studies. The results were visualized in forest plots. Subgroup analyses were used to address heterogeneity and variability in the dependent variable and age of patients in the control and experimental groups. Heterogeneity between studies was assessed by using the *I*^2^ test, the chi-squared test and forest plots. Heterogeneity was considered statistically significant when the *P* value < 0.05 or *I*^*2*^ values > 50% [33,34]. A random-effects model was used when heterogeneity was considered; otherwise, a fixed-effects model was used. In addition, sensitivity analyses were used to assess the robustness.

## Result

### General characteristics of the included studies

**Fig 1:**
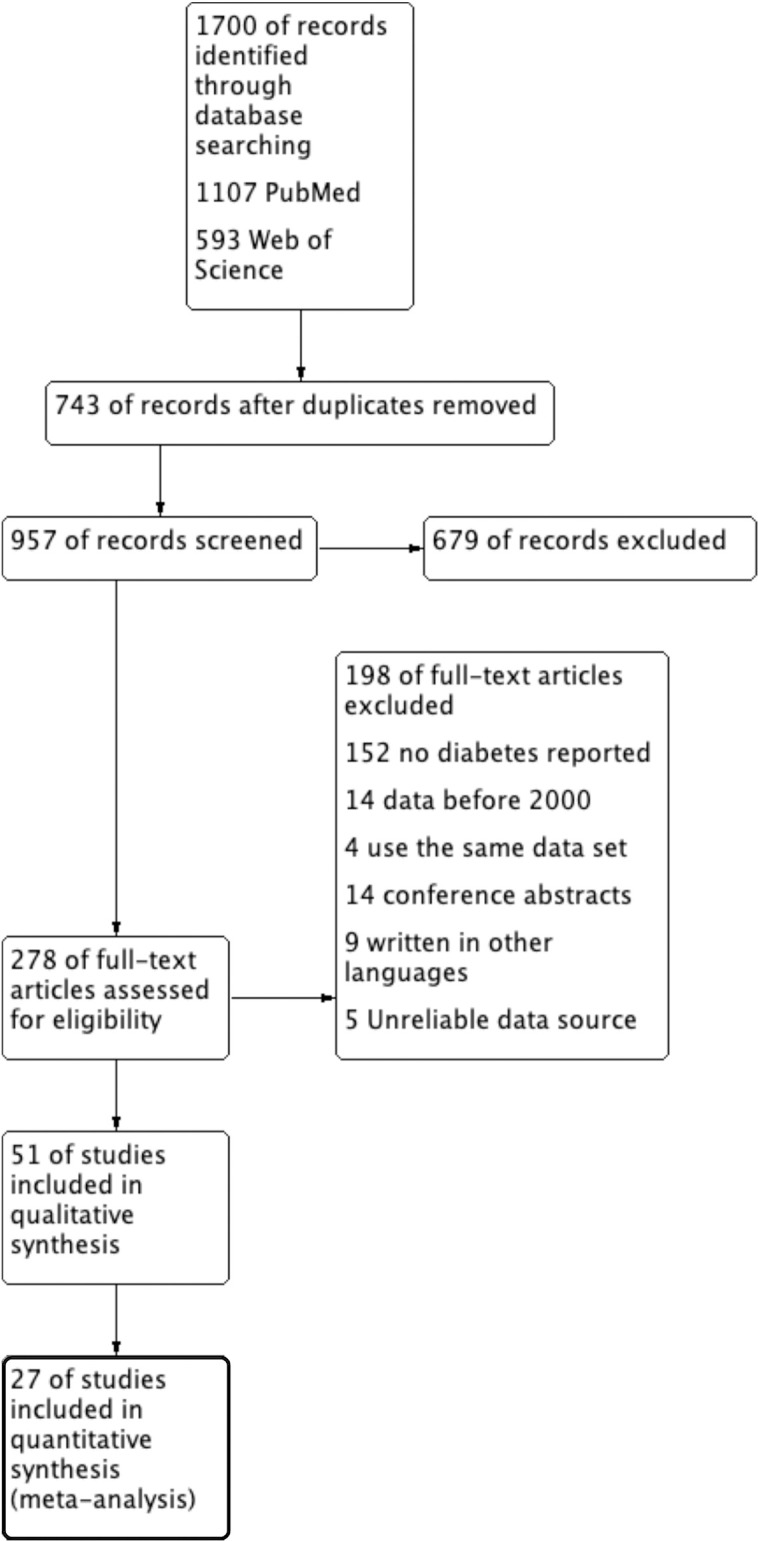
Flow diagram of publication selection process.

In total, 1700 studies were retrieved from the database in the initial search, of which 278 were considered potentially eligible after reviewing the titles and abstracts. After reading the full text of the articles, 31 articles were eligible, of which 4 studies used the same dataset as the others, and 27 articles were included in this meta-analysis [15,20,25–31,35–52]. The processes of study screening are shown in Fig 1, and the general characteristics of the included studies and the corresponding NOS scores are shown in Tables 1 and 2. Of these 27 studies, 19 were studies on dengue, 1 study was only able to extract data on deaths [49], and the remaining 8 were studies on WNF. In addition, most of these studies were case–control studies based on hospital administrative records. Both dengue and WNF were diagnosed by ELISA or RT–PCR, whereas DM was mostly derived from case records or self-reported by patients, and nine of these studies did not report the source of the DM diagnosis. Seven studies on dengue defined severe illness according to the WHO 1997 criteria, 7 studies defined it according to the WHO 2009 criteria, and the remaining studies combined both criteria. Subsequently, we divided the severe cases of both diseases into DHF (8 studies) and DSS (2 studies) according to the WHO 1997 criteria, and the remaining 8 studies used the WHO 2009 criteria and therefore were not included in the subgroup analysis. WNF was classified as WNM (1 study), WNE (2 studies), and WNVR (1 study). Finally, we tried to extract mortality data to analyze the effect of DM on mortality. We extracted 10 studies from 27 studies, 6 studies on dengue and 4 studies on WNF(S1 Table).

**Table 1:** Characteristics of the studies included in the meta-analysis.

Authors (publication date)	Area	Study desigh	Classification	Case identification methods		NOS score	Included in meta-analysis
				Dengue fever/West Nile fever	diabetes mellitus		
Jisamerin et al. (2021) [41]	Tamil Nadu, India	Retrospective	2009 WHO Classification	ELISA	Report review	6	Yes
Chen et al. (2015) [39]	Gaoxiong, Taiwan	Retrospective	1997 WHO Classification	ELISA	Report review	9	Yes
Blanco et al. (2018) [38]	Tanzania	Retrospective	2009 WHO Classification	ELISA	/	7	Yes
Figueiredo et al. (2010) [40]	Brazilian	case–control	1997 WHO Classification	ELISA	/	8	Yes
Wang et al. (2019) [50]	Kaohsiung and Tainan, Taiwan	Retrospective	1997 WHO Classification	ELISA	/	9	Yes
Banni et al. (2020) [35]	Jeddah, Saudi Arabia	case–control	2009 WHO Classification	ELISA	/	6	Yes
Mallhi et al. (2015) [46]	Kelantan, Malaysia	Retrospective	1997 WHO Classification	RT–PCR+ ELISA	Report review	8	Yes
Thein et al. (2014) [49]	Singaporean	case–control	Both 1997 and 2009 WHO Classification	ELISA	Report review	8	Yes
Thein et al. (2013) [53]	Singaporean	case–control	2009 WHO Classification	ELISA	Report review	8	NO
Karunakaran et al. (2014) [42]	Kerala, India	case–control	2009 WHO Classification	ELISA	Report review	9	Yes
Thein et al. (2016) [54]	Singaporean	case–control	2009 WHO Classification	ELISA	Report review	8	NO
Wei et al. (2016) [51]	Taiwan	case–control	Both 1997 and 2009 WHO Classification	RT–PCR+ ELISA	Report review	7	Yes
Han et al. (2017) [47]	Kuala Lumpur, Malaysia	case–control	Both 1997 and 2009 WHO Classification	ELISA	Report review	6	Yes
Kuo et al. (2017) [43]	Gaoxiong, Taiwan	case–control	2009 WHO Classification	ELISA	Report review	8	Yes
Agrawal et al. (2018) [36]	Hyderabad, India	case–control	2009 WHO Classification	ELISA	On-site testing	6	Yes
Salim et al. (2012) [20]	Singaporean	case–control	1997 WHO Classification	RT–PCR+ ELISA	Report review	7	Yes
Mirza et al. (2016) [37]	Lahore, Pakistan	Retrospective	Both 1997 and 2009 WHO Classification	ELISA	/	8	Yes
Lee et al. (2006) [45]	Taiwan	case–control	1997 WHO Classification	RT–PCR+ ELISA	Report review	7	Yes
Wei et al. (2022) [48]	Malaysia	case–control	2009 WHO Classification	ELISA	/	8	Yes
Werneck et al. (2018) [52]	Brazilian	retrospective cohort	1997 WHO Classification	ELISA	/	6	Yes
Lee et al. (2020) [44]	Gaoxiong, Taiwan	case–control	Both 1997 and 2009 WHO Classification	ELISA	Report review	8	Yes
Bode et al. (2006) [31]	Colorado, USA	Retrospective	WNM+WNE	RT–PCR+ ELISA	Report review	9	Yes
Staples et al. (2012) [27]	USA	Retrospective	WNND	ELISA	Report review	8	Yes
Racsa et al. (2014) [26]	Texas, USA	Retrospective	WNND	ELISA	/	6	Yes
Baraniuk et al. (2006) [30]	Houston, Texas, USA	nested case control	WNE	ELISA	Report review	7	Yes
Murray et al. (2008) [55]	Houston, Texas, USA	case–control	WNE	ELISA	Report review	6	NO
Weatherhead et al. (2015) [56]	Houston, Texas, USA	case–control	WNE	ELISA	Report review	7	NO
Jean et al. (2007) [29]	California, USA	Retrospective	WNND	ELISA	Report review	8	Yes
Snyder et al. (2020) [15]	California, USA	Retrospective	/	RT–PCR+ ELISA	Report review	7	Yes
Khairallah et al. (2007) [28]	Monastir, Tunisia	Retrospective	WNVR	ELISA	Report review	6	Yes
Vrioni et al. (2014) [25]	Greece	case–control	WNND	ELISA	/	6	Yes

ELISA (Enzyme-Linked Immunosorbent Assays): A qualitative and quantitative detection method for immune reactions by binding soluble antigens or antibodies to solid phase carriers and utilizing antigen antibody binding specificity; RT–PCR (Real-time polymerase chain reaction): A molecular biology technique for real-time amplification and detection of specific DNA or RNA sequences. WNM: West Nile meningitis; WNE: West Nile encephalitis; WNND: West Nile Neuroinvasive Disease; WNVR: West Nile virus-associated retinopathy.

**Table 2:** The qualities of the included studies were assessed by the Newcastle Ottawa Quality Scale (NOS).

	Case definition	Case representativeness	Selection of controls	Definition of comparison	Comparability between case and control	Determination of exposure	Is exposure determined using the same method	Nonresponse rate	score
Jisamerin et al. (2021) [41]	*	/	/	*	*	**	*	/	6
Chen et al. (2015) [39]	*	*	/	*	**	**	*	*	9
Blanco et al. (2018) [38]	*	/	/	*	**	**	*	/	7
Figueiredo et al. (2010) [40]	*	*	*	*	**	*	*	*	8
Wang et al. (2019) [50]	*	*	/	*	**	**	*	*	9
Banni et al. (2020) [35]	*	*	*	*	/	/	*	*	6
Mallhi et al. (2015) [46]	*	*	/	*	**	*	*	*	8
Thein et al. (2014) [49]	*	*	/	*	**	*	*	*	8
Thein et al. (2013) [53]	*	*	*	*	**	*	*	*	9
Karunakaran et al. (2014) [42]	*	*	*	*	*	*	*	*	8
Thein et al. (2016) [54]	/	/	/	*	**	*	*	*	6
Wei et al. (2016) [51]	*	/	/	*	*	*	*	/	5
Han et al. (2017) [47]	*	*	/	*	/	**	*	*	7
Kuo et al. (2017) [43]	*	*	/	*	**	**	*	*	9
Agrawal et al. (2018) [36]	/	*	/	*	**	*	*	/	6
Salim et al. (2012) [20]	*	*	/	*	**	*	*	*	8
Mirza et al. (2016) [37]	*	*	/	*	*	*	*	/	6
Lee et al. (2006) [45]	*	*	/	*	*	*	*	*	7
Wei et al. (2022) [48]	*	*	/	*	**	*	*	*	8
Werneck et al. (2018) [52]	*	/	/	*	*	*	*	*	6
Lee et al. (2020) [44]	*	*	/	*	**	*	*	*	8
Bode et al. (2006) [31]	*	*	/	*	**	**	*	*	9
Staples et al. (2012) [27]	*	*	/	*	**	*	*	*	8
Racsa et al. (2014) [26]	*	*	/	*	/	*	*	*	6
Baraniuk et al. (2006) [30]	*	/	*	*	*	*	*	*	7
Murray et al. (2008) [55]	*	/	/	*	**	*	*	/	6
Weatherhead et al. (2015) [56]	*	/	/	*	**	*	*	*	7
Jean et al. (2007) [29]	*	/	*	*	**	*	*	*	8
Snyder et al. (2020) [15]	*	/	/	*	**	*	*	*	7
Khairallah et al. (2007) [28]	*	*	/	*	/	*	*	*	6
Vrioni et al. (2014) [25]	*	*	/	*	/	*	*	*	6

*: get a point in this question;/: no point in this question.

### Risk of bias assessment

All 31 studies were evaluated by the NOS tool. Five data points scored 9, nine data points scored 8, four data points scored 7, and eight data points scored 6. The findings of the funnel plot were confirmed by Egger’s test, indicating no significant publication bias in the analysis except for the results of two studies on dengue (*P*>0.05) (Table 3).

**Table 3:** Summary of combined impact estimates related to dengue and West Nile fever.

Disease	Outcomes	No. of studies	Test of association		Test of heterogeneity			Egger’s test P value
			OR	95% CI	χ^2^	I^2^(%)	P value	
Dengue	Entire	18	3.39	2.46, 4.68	70.60	76%	<0.001	0.006
	DHF	8	2.73	1.68, 4.44	75.18	86%	<0.001	0.032
	DSS	2	7.29	3.09, 17.20	0.15	0%	0.703	/
	Death	6	1.95	1.09, 3.52	9.02	45%	0.108	0.813
West Nile fever	WNND	8	2.89	1.89, 4.41	21.76	67%	0.003	0.375
	WNM	1	1.15	0.38, 3.49	/	/	/	/
	WNE	2	3.29	1.15, 9.40	2.66	62%	0.103	/
	WNVR	1	11.00	1.13, 106.84	/	/	/	/
	Death	4	1.74	1.40, 2.17	1.80	0%	0.616	0.563

DHF: dengue hemorrhagic fever; DSS: dengue shock syndrome; WNM: West Nile meningitis; WNE: West Nile encephalitis; WNND: West Nile Neuroinvasive Disease; WNVR: West Nile virus-associated retinopathy.

### Effects of DM on dengue and WNF

**Fig 2:**
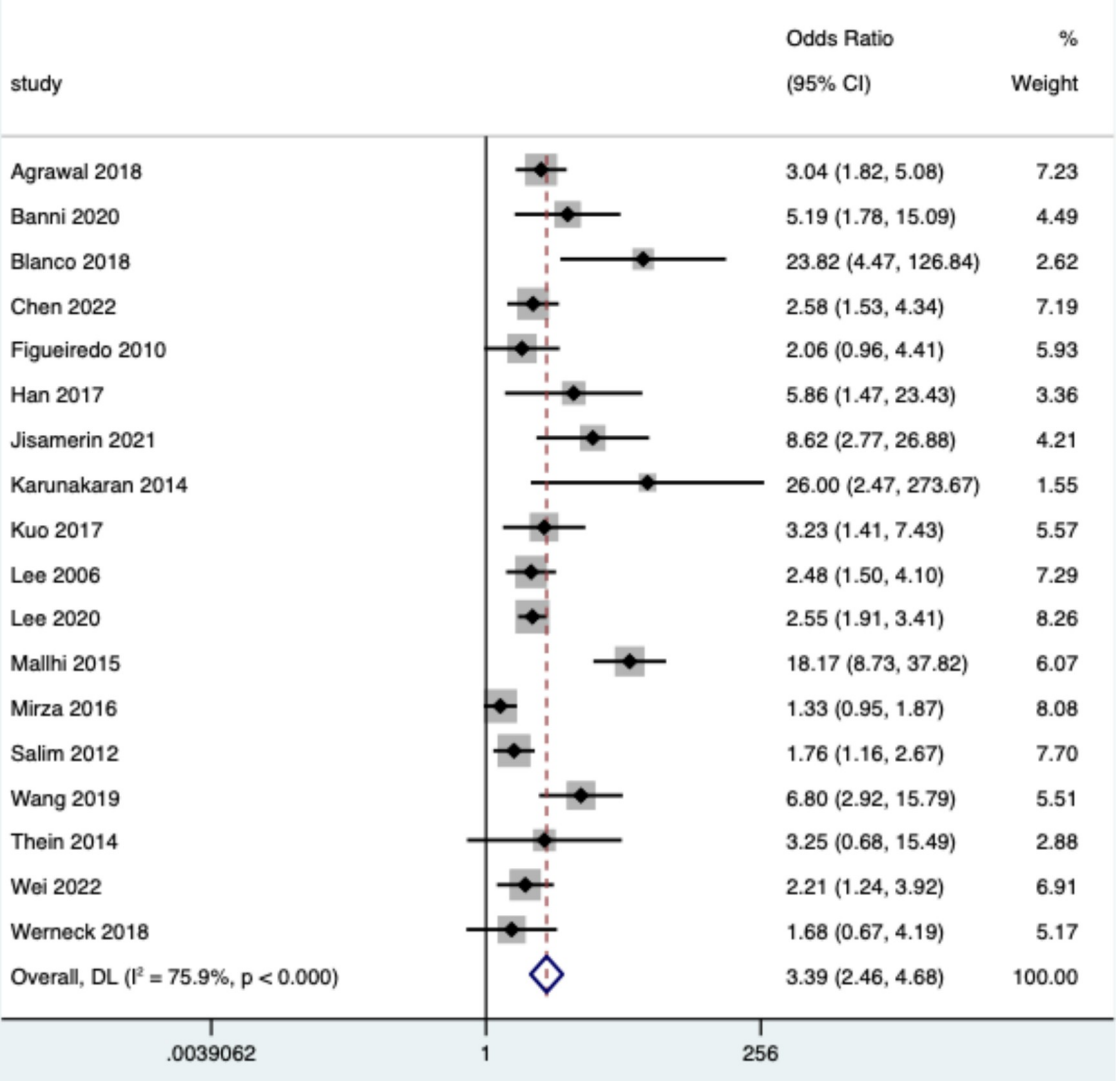
Forest plot of dengue. The size of the black square corresponding to each study is proportional to the sample size, and the center of each square represents the OR. Horizontal line shows the corresponding 95% CI of the OR. Pooled OR is represented by hollow diamond.

**Fig 3:**
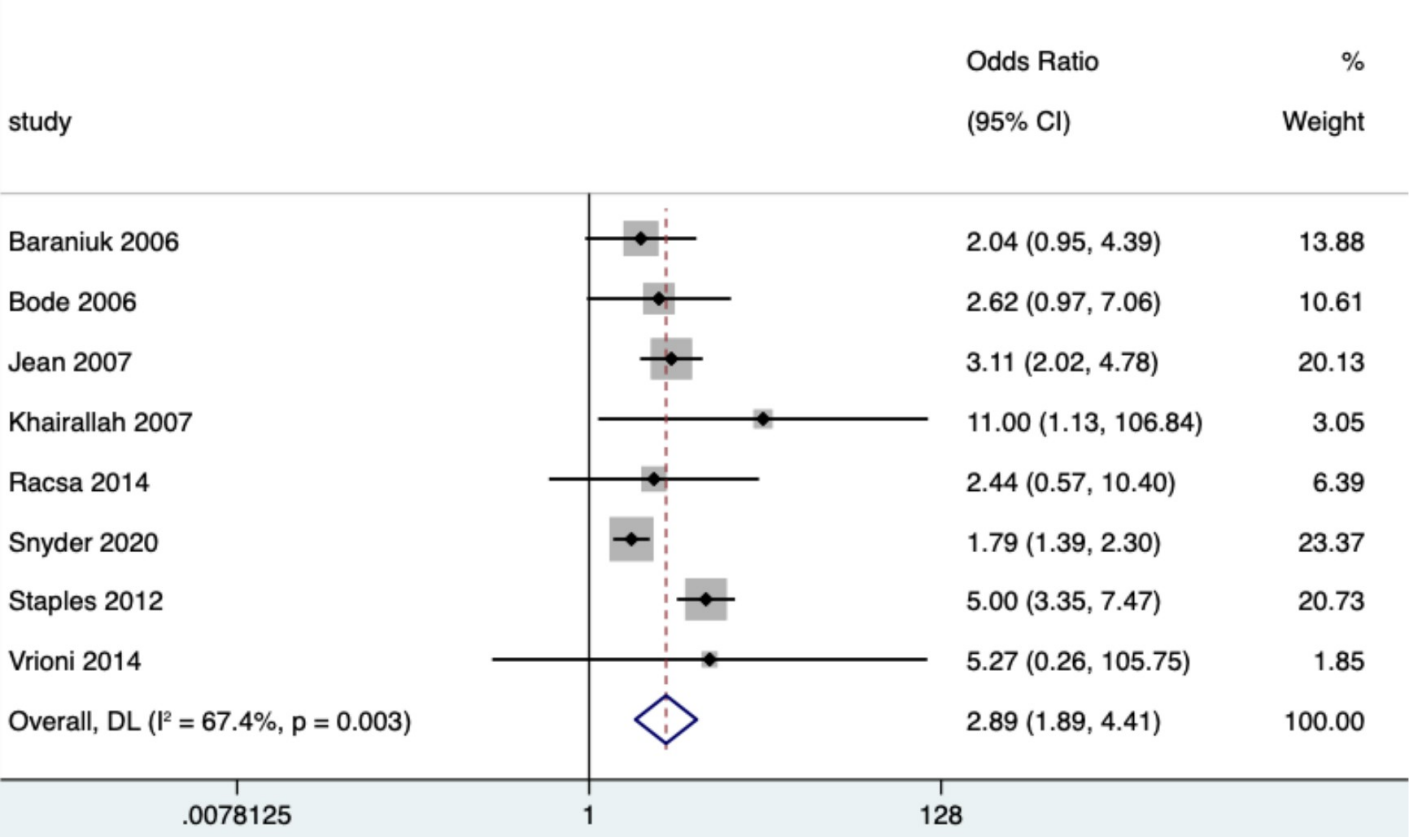
Forest plot of West Nile fever. The size of the black square corresponding to each study is proportional to the sample size, and the center of each square represents the OR. Horizontal line shows the corresponding 95% CI of the OR. Pooled OR is represented by hollow diamond.

Eighteen and 8 studies reported the effect of DM on dengue and West Nile fever severity, respectively. Heterogeneity tests showed a high degree of heterogeneity in the effect of DM on the two diseases, with *I*^2^(*P*) values of 76% (*P* < 0.001) and 66% (*P* = 0.003), respectively. Therefore, a random effects model was used to estimate the combined effect of DM on both diseases. The results showed that DM significantly increased the risk of severe dengue and WNF, with ORs of 3.39 (95% CI: 2.46, 4.68) and 2.89 (95% CI: 1.89, 4.41), respectively (Table 3 and Figs 2 and 3). The funnel plot showed publication bias (*P*<0.05) on the effect of DM on dengue fever, while there was no publication bias (*P*<0.05) on WNF (Table 3 and S1, S2, S3 and S4 Figs). Sensitivity analysis showed a significant change in the study results when two articles, Mallhi (2015) and Mirza (2016), were excluded, and the heterogeneity was reduced from 76% to 47%.

**Fig 4:**
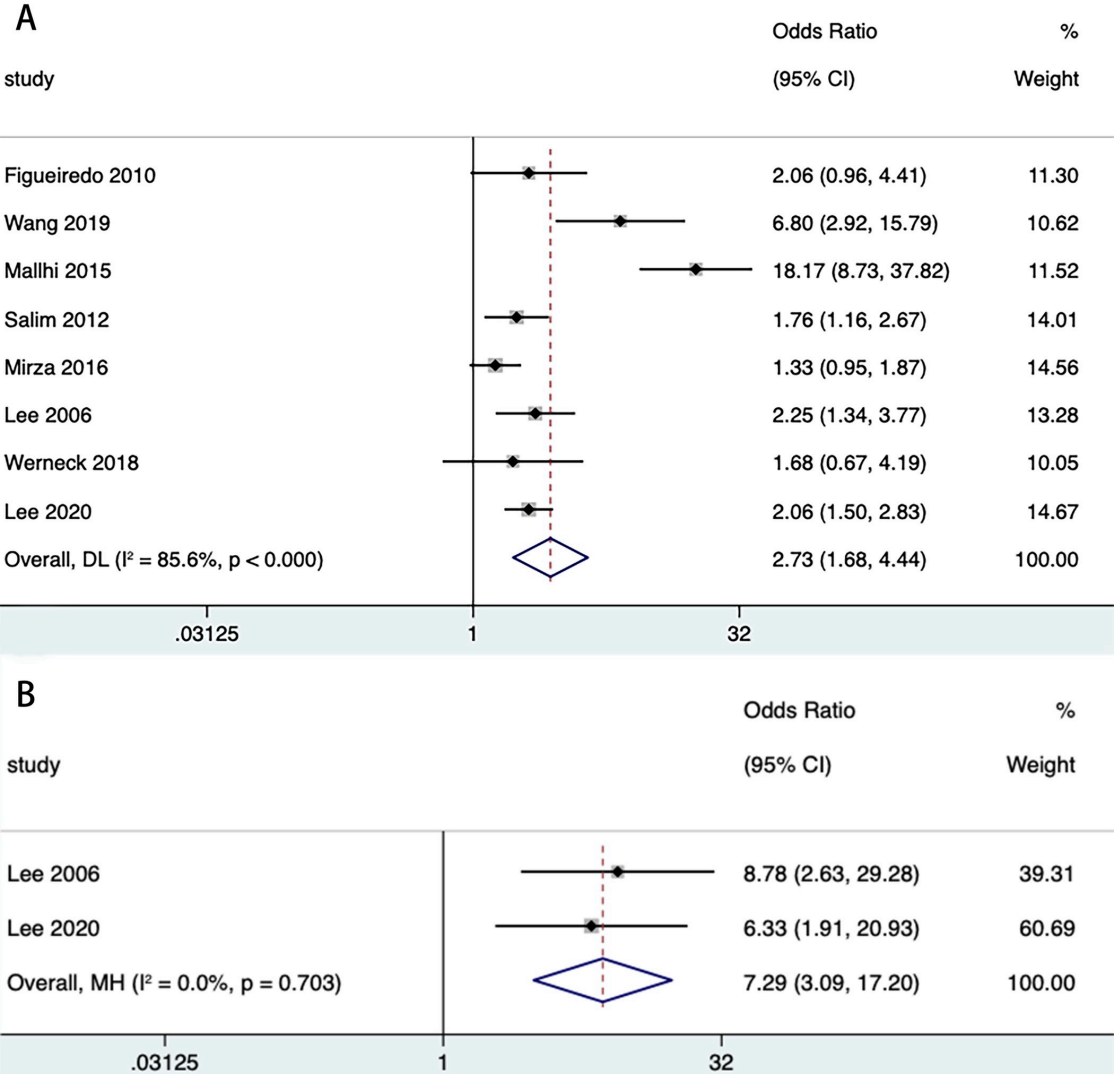
Forest plot of dengue disease process according to the 1997 WHO criteria. **Forest plot of denguehemorrhagic fever (A). Forest plot of dengue shock syndrome (B).** The size of the black square corresponding to each study is proportional to the sample size, and the center of each square represents the OR. Horizontal line shows the corresponding 95% CI of the OR. Pooled OR is represented by hollow diamond.

**Fig 5:**
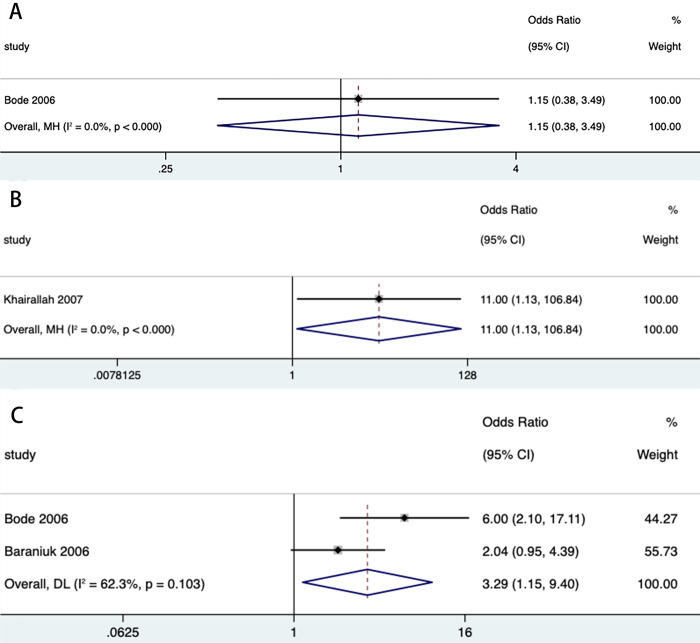
Forest plot of severe West Nile fever with different manifestations. **Forest plot of West Nile meningitis (A). Forest plot of West Nile encephawlitis (B). Forest plot of West Nile virus-associated retinopathy (C).** The size of the black square corresponding to each study is proportional to the sample size, and the center of each square represents the OR. Horizontal line shows the corresponding 95% CI of the OR. Pooled OR is represented by hollow diamond.

**Fig 6:**
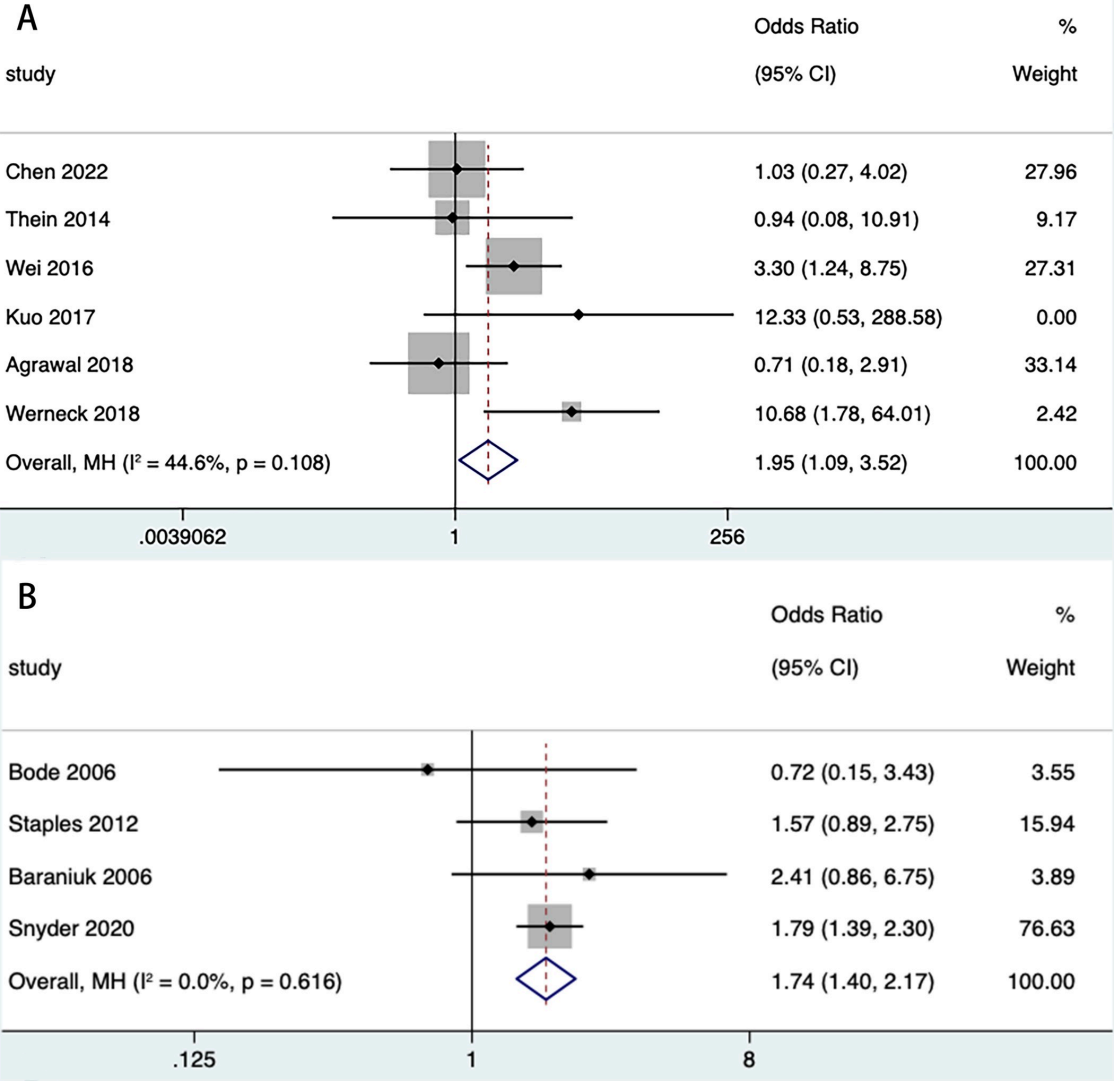
Forest plot of death. **Forest plot of diabetes mellitus on death of dengu (A). Forest plot of diabetes mellitus on death of West Nile fever (B).** The size of the black square corresponding to each study is proportional to the sample size, and the center of each square represents the OR. Horizontal line shows the corresponding 95% CI of the OR. Pooled OR is represented by hollow diamond.

We then stratified dengue (DHF, DSS) and WNF (WNM, WNE, WNVR) by disease progression or clinical symptoms to estimate the effect of DM on both. The combined effect of DM on DHF (n = 8) (OR = 2.73, 95% CI: 1.68, 4.44) and DSS (n = 2) (OR = 7.29, 95% CI: 3.09, 17.20) was statistically significant in both classifications of dengue (Table 3 and Fig 4). Among the 3 classifications of WNF, the combined effect of DM on WNE (n = 2) (OR = 3.29, 95% CI: 1.15, 9.40) and WNVR (n = 1) (OR = 11.00, 95% CI: 1.13, 106.84) was statistically significant, whereas WNM (n = 1) (OR = 1.15, 95% CI: 0.38, 3.49) did not show statistical significance (Table 3 and Fig 5). We then extracted mortality data for subgroup analyses to analyze the impact of DM on mortality from both diseases. The results showed a statistically significant combined effect of DM on dengue deaths (n = 6) (OR = 1.95, 95% CI: 1.09, 3.52) and West Nile fever deaths (n = 4) (OR = 1.74, 95% CI: 1.40, 2.17) (Table 3 and Fig 6). With the exception of all studies (*P* = 0.006) and DHF (*P* = 0.032), the funnel plot did not show publication bias in several stratified studies (*P*>0.05) (S5, S6, S7, S8, S9 and S10 Figs).

## Discussion

In this meta-analysis, we found that having DM was a demographic risk factor for the progression of DF and WNF to severe disease. Additionally, the results of the subgroup analyses showed that differences in the dependent variable (deterioration or death) did not lead to changes in the conclusions.

Our study also suffers from a number of shortcomings that cannot be addressed at this time. Most of the studies selected were retrospective, had different clinical and laboratory diagnostic criteria and control groups and were heterogeneous in terms of exposure and outcome. Some studies only used ELISA for diagnosis, which may result in serological cross-reactivity [57]. Most patients with DM have other comorbidities (e.g., hypertension, coronary artery disease), and we were unable to assess the impact of DM on the conditions of DF and WNF in isolation. At the same time, the judgment and inclusion criteria of DM in these articles are not clear, and only three studies showed a specific typology of DM (type 2) [39,43,44]. The diagnosis of DM mostly comes from case records, and some patients report themselves. It is difficult to distinguish whether DM is diagnosed before, after or during the onset of infection. Based on the literature included, we found that the majority of WNF studies originated in the United States, which may be related to the repeated outbreaks of WNF in North America over the past decade, which have resulted in the continued spread of WNF in communities [58–60]. This strong geographic trend may lead to impeded extrapolation of results. Most of the studies in dengue have adopted the new case classification of the WHO in 2009 to improve case management in the clinical setting. However, a proportion of reports still used the 1997 version, which was developed by the WHO based on a model of DF in Thai children and lacked clarity in the description of the measurement of outcome endpoints [61].

DM has been listed as a significant predictor of flavivirus-caused disease in the past, and this is supported by the findings of our meta-analysis. The underlying pathophysiologic mechanisms regarding the role of DM in the progression of DF and WNF are not completely clear at this time. Lee et al. found that patients with difficult glycemic control (HbA1c >7%) had a higher risk of dengue exacerbation than diabetic patients with better glycemic control (both without additional comorbidities) [44]. Hyperglycemia in diabetic patients is thought to lead to immune response dysfunction, with suppression of cytokine production, defective phagocytosis, and immune cell dysfunction, in addition to the risk of natural barrier impairment due to neuropathy [62,63]. This provides more opportunities for viruses to invade. In addition, platelet activity is increased to varying degrees in both type 1 and type 2 diabetic patients [64]. Platelets can interact with neutrophils to promote their activation and release of platelet factor (CXCL4), which has been shown to significantly inhibit the interferon pathway and enhance DENV replication in cells both in vitro and in vivo [65]. In addition, there is some biological evidence that can prove that patients with DM who are infected with DENV and WNV are more likely to aggravate infection. A study has shown that blood sugar is conducive to DENV replication, and it promotes virus transmission in mosquitoes through AKT and TOR signaling [66]. Furthermore, the study reveals that mosquito cells incubated in a high glucose medium exhibit upregulated levels of DENV proteins NS1, NS5, E, and prM, as well as AKT signaling (AKT phosphorylation) and TOR signaling (S6K phosphorylation) [66]. Some articles have found that monocytes infected with DENV in type 2 DM increase the production of interleukin-4 (IL-4), interleukin-10 (IL-10) and granulocyte-macrophage colony stimulating factor (GM-CSF) [67]. According to records, T helper (Th) cells play an important role in the immune pathogenesis of DHF [68]. According to the type of cytokine produced during activation, Th cells are divided into Th1 and Th2 cells. Activated Th1 cells produce IFN-γ (interferon), IL-2 and IL-12, while Th2 cells produce IL-4, IL-5, IL-10 and IL-13 [69]. Among the various mechanisms of the pathogenesis of DF, it has been reported that in secondarily infected hosts, a high DENV load is indirectly associated with DHF [70], and the overwhelming activation of Th2 cytokines has been documented in the development of DHF in patients with primary and secondary infections with DF [68,71]. Specifically, in the Th2 cytokine spectrum, IL-4 is the most effective cytokine for inducing Th2 cell differentiation, and IL-10 is responsible for the anti-inflammatory response in host immune activity [72]. In addition, it has been reported that compared with mild DF patients, the serum GM-CSF of severe DF patients is significantly increased [73]. Our stratified analysis similarly confirms this observation, with the OR value for DSS (7.29) being significantly higher than that for DHF (2.73).

Mukesh Kumar and others performed experiments on WNV infection in diabetic mouse models. The diabetic mouse model showed a high susceptibility to WNV disease, showing higher tissue tropism and mortality than wild-type mice. This is related to WNV infection and increased inflammation in diabetic mice and severely impaired and delayed specific immune response, which is characterized by delayed induction of IFN-α (interferon), and the concentration of WNV-specific IgM and IgG antibodies decreased in viremia [74]. Later, they discovered that the presence of DM significantly changed the recruitment of white blood cells in the brain, resulting in failure to clear the WNV infection in the brains of diabetic mice [75]. These findings are consistent with our study, emphasizing the importance of researching the role of DM in DF and WNF infections, and highlighting that DM could worsen the symptoms and severity of these diseases. Thus, our study further supports the need to focus on DM in the management of DF and WNF infections, as well as the importance of individualized intervention measures for patients with DM.

In addition to dengue and WNF, several studies have shown the effect of DM on Zika virus disease, Japanese encephalitis, and yellow fever. Azar et al. demonstrated increased susceptibility of *Aedes aegypti* that fed on “diabetic” bloodmeals to ZIKV by in vitro and in vivo modeling of type II DM and suggested that the prevalence of type II DM in the population may have a significant impact on ZIKV transmission [76]. Ahlers et al. showed that mammalian insulin can trigger AKT and ERK signaling in mosquitoes, leading to the transcription of JAK/STAT-associated antiviral genes [77]. DM was one of the most common comorbidities in the study patients (9.94%), and patients with comorbid JEV had higher medical costs than patients without DM [78]. Studies have shown that JEV comorbid with DM significantly increased the risk of death by 2.47 times (*P*<0.05) [74]. Our results indicate that diabetes has a statistically significant combined impact on dengue fever mortality (OR = 1.95) and West Nile fever mortality (OR = 1.74). Patients with YFV and DM had a higher case fatality rate (CFR) of 80% compared with 65% in patients without DM [79]. In addition, DM attenuates the YFV vaccine effect by reducing 2’,5’-oligoadenylate synthase levels. Basal 2’,5’-oligoadenylate activity increased several-fold in response to YFV vaccination. In DM subjects, this increase was significantly lower (*P* = 0.025) [80]. Based on these reports, DM can be shown to increase the risk of adverse outcomes of mosquito-borne flavivirus infection [14,76,80].

Overall, studying DM for DF and WNF infections is important to reduce the burden of disease by guiding approaches to improve patient prognosis or differential case management. We provide evidence that the prevalence of DM is higher in severe cases of dengue and WNF infection than in nonsevere cases. This means that DM may exacerbate the symptoms of DF and WNF infections. A further study with more focus on DM, DENV and WNV is therefore suggested. Examples include longitudinal studies of DM, DF and WNF, i.e., the effect of blood glucose concentration on the clinical symptoms of the disease. In addition, standardized prospective cohort studies in areas with high rates of infection will help to better understand the etiological role of DM in serious disease outcomes and to evaluate the causal relationship between them. This study can also provide some warnings for doctors who have DENV or WNV patients. For example, when a DENV or WNV patient with DM appears, the doctor should promptly decide whether it needs close observation, adequate treatment or hospitalization, and when a patient with DF has severe clinical symptoms, the doctor should promptly ask about past medical history, especially the history of DM. Patients with DM living in areas with high rates of DENV and WNV infection should be given a higher level of attention after diagnosis.

## Supporting information

S1 Fig Egger’s test of dengue. (TIF)

S2 Fig Trim and fill analysis of dengue. (TIF)

S3 Fig Egger’s test of West Nile fever. (TIF)

S4 Fig Trim and fill analysis of West Nile fever. (TIF)

S5 Fig Egger’s test of dengue hemorrhagic fever. (TIF)

S6 Fig Trim and fill analysis of dengue hemorrhagic fever. (TIF)

S7 Fig Egger’s test of death of dengue. (TIF)

S8 Fig Trim and fill analysis of death of dengue. (TIF)

S9 Fig Egger’s test of death of West Nile fever. (TIF)

S10 Fig Trim and fill analysis of death of West Nile fever. (TIF)

S1 Table Data collection of the studies included in the meta-analysis. (DOCX)
