# Supplementary figures and images for: Diabetes mellitus as a risk factor for severe dengue fever and West Nile fever: A meta-analysis
Source: PLoS Negl Trop Dis. 2024 May 31;18(5):e0012217. doi: 10.1371/journal.pntd.0012217 (PMC11168630; doi:10.1371/journal.pntd.0012217)

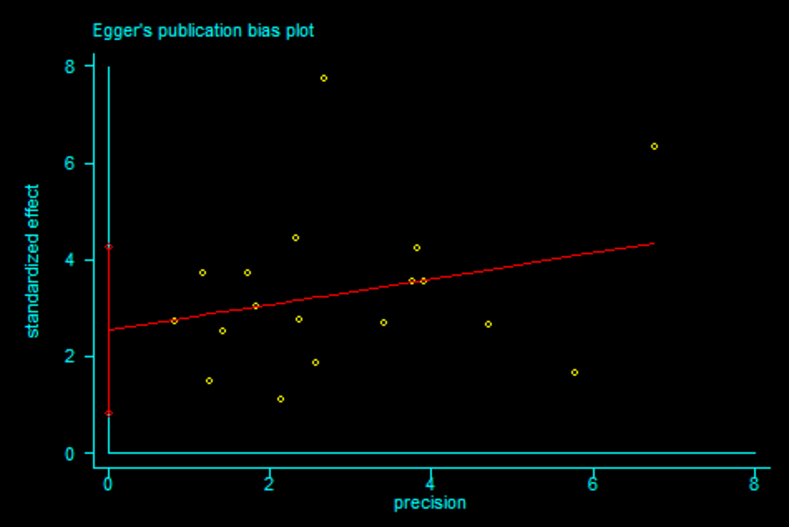

Supplement: S1 Fig — (TIF) [file pntd.0012217.s001.tif]

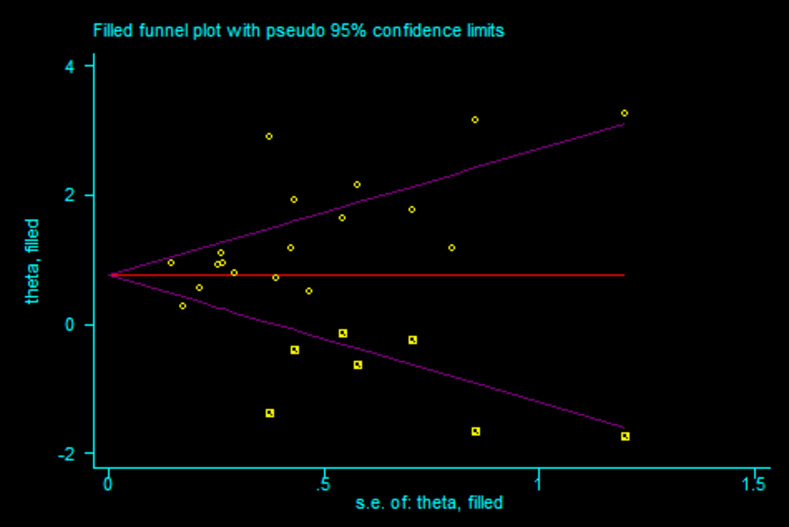

Supplement: S2 Fig — (TIF) [file pntd.0012217.s002.tif]

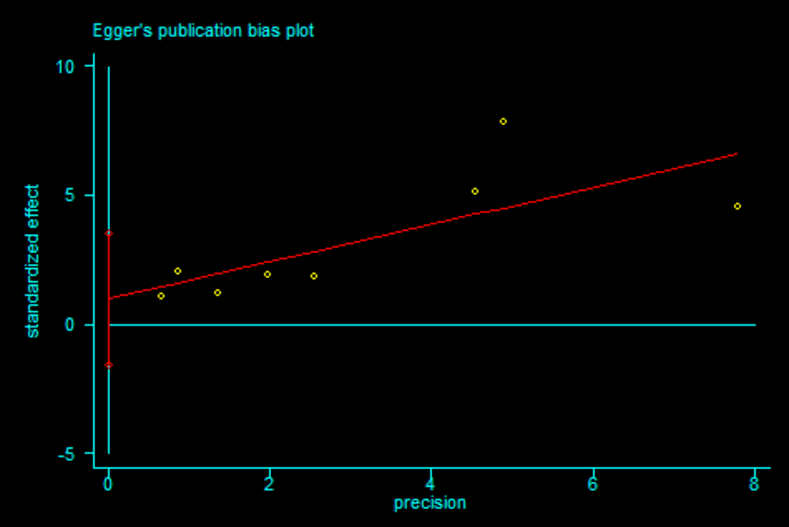

Supplement: S3 Fig — (TIF) [file pntd.0012217.s003.tif]

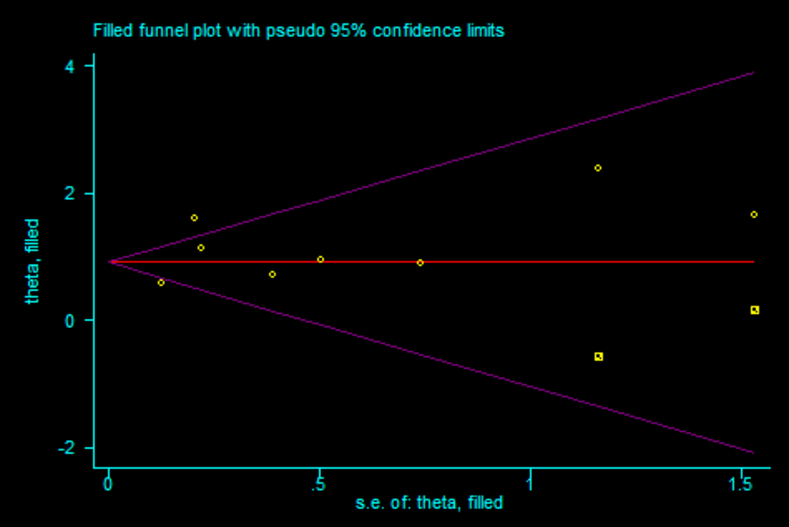

Supplement: S4 Fig — (TIF) [file pntd.0012217.s004.tif]

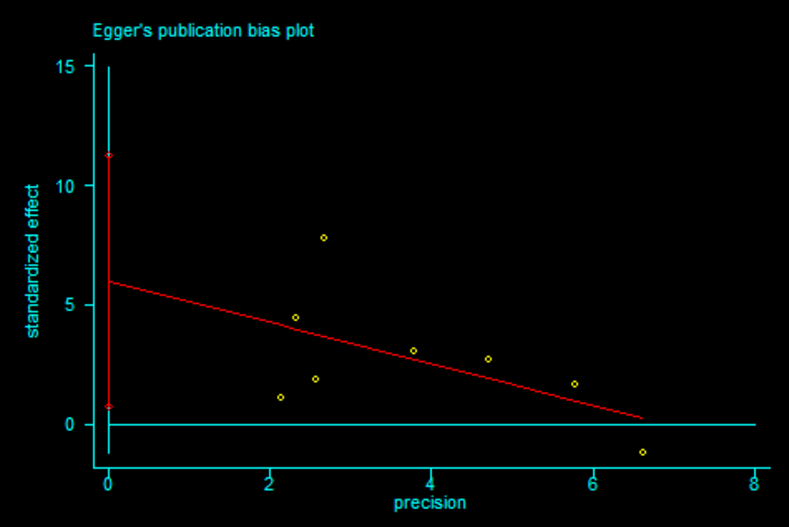

Supplement: S5 Fig — (TIF) [file pntd.0012217.s005.tif]

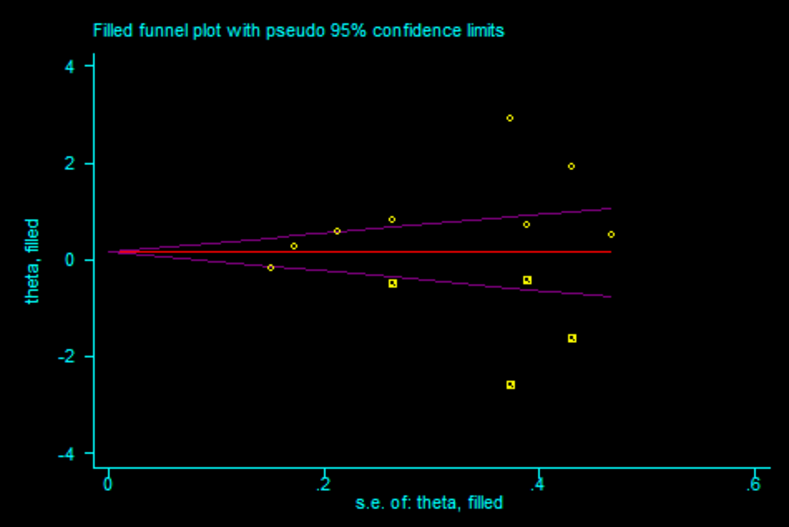

Supplement: S6 Fig — (TIF) [file pntd.0012217.s006.tif]

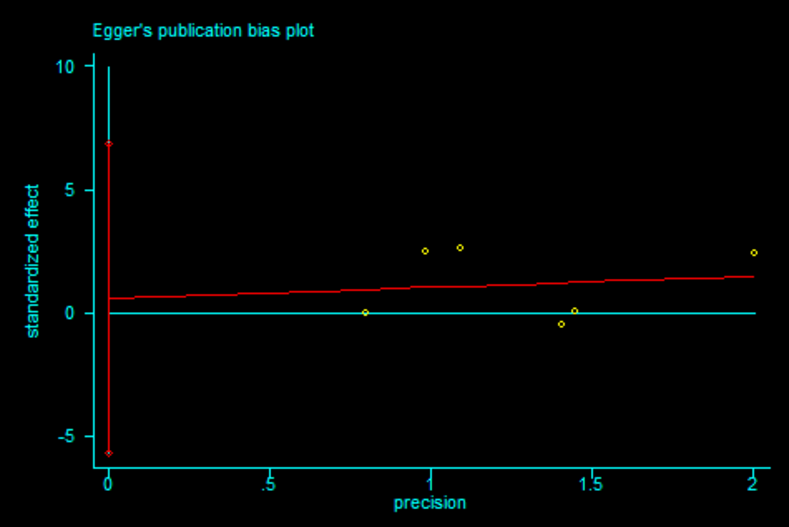

Supplement: S7 Fig — (TIF) [file pntd.0012217.s007.tif]

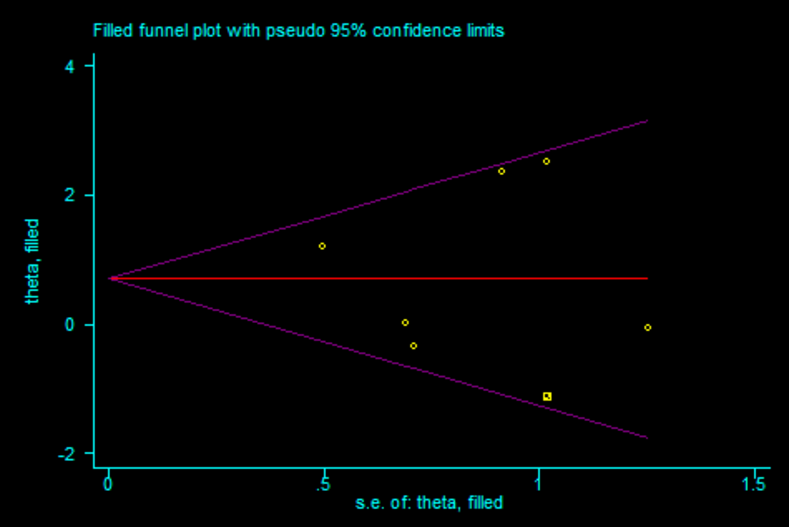

Supplement: S8 Fig — (TIF) [file pntd.0012217.s008.tif]

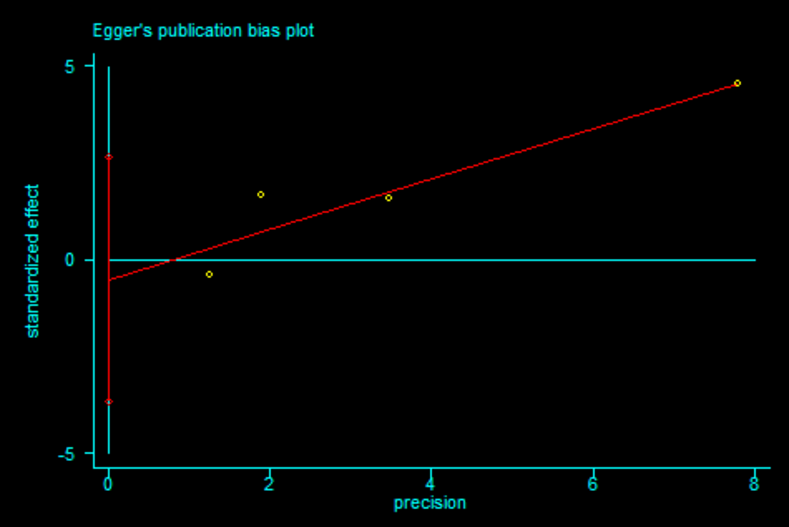

Supplement: S9 Fig — (TIF) [file pntd.0012217.s009.tif]

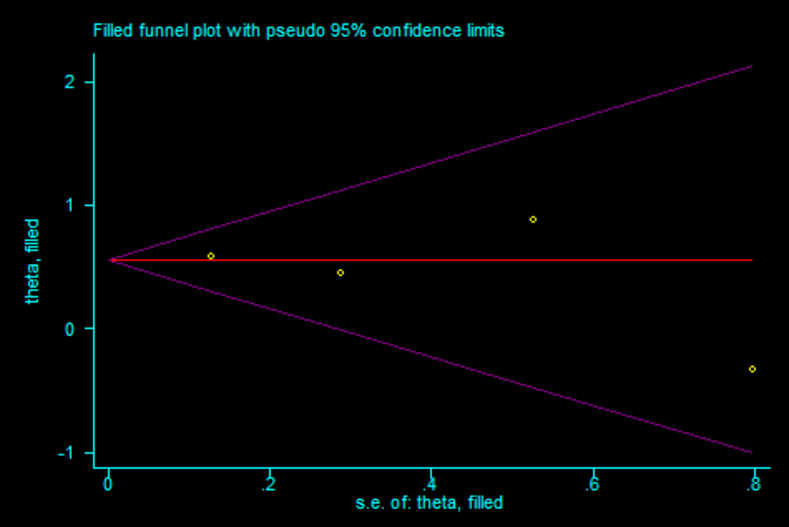

Supplement: S10 Fig — (TIF) [file pntd.0012217.s010.tif]
