# Supplementary material for: Diabetes mellitus as a risk factor for severe dengue fever and West Nile fever: A meta-analysis
Source: PLoS Negl Trop Dis. 2024 May 31;18(5):e0012217. doi: 10.1371/journal.pntd.0012217 (PMC11168630; doi:10.1371/journal.pntd.0012217)
Supplement: S1 Table — (DOCX) [file pntd.0012217.s011.docx]

**S1 Table Data collection of the studies included in the meta-analysis**

| Authors  (publication date) | Area | Study desigh | Case identification methods | | | | Total number of cases | severe cases | Non-severe cases | Diabetes patients in severe group | Diabetes patients in non-severe group |
| --- | --- | --- | --- | --- | --- | --- | --- | --- | --- | --- | --- |
|  |  |  | Dengue fever/West Nile fever | diabetes mellitus | | |  |  |  |  |  |
| Jisamerin et al.  (2021) (1) | Tamil Nadu, India | Retrospective | ELISA | | Report review | 150 | | 29 | 121 | 9 | 6 |
| Chen et al.  (2015) (2) | Gaoxiong, Taiwan | Retrospective | ELISA | | Report review | 644 | | 233(DHF and DSS) | 411(DF) | 37 | 28 |
| Blanco et al.  (2018) (3) | Tanzania | Retrospective | ELISA | | / | 428 | | 20 | 408 | 3 | 3 |
| Figueiredo et al.  (2010) (4) | Brazilian | case-control | ELISA | | / | 1345 | | 170(DHF) | 1175(DF) | 9 | 31 |
| Wang et al.  (2019) (5) | Kaohsiung and Tainan, Taiwan | Retrospective | ELISA | | / | 135 | | 45(DHF) | 90(DF) | 23 | 12 |
| Banni et al.  (2020) (6) | Jeddah, Saudi Arabia | case-control | ELISA | | / | 368 | | 123 | 245 | 12 | 5 |
| Mallhi et al.  (2015) (7) | Kelantan, Malaysia | Retrospective | RT-PCR+ ELISA | | Report review | 667 | | 79(DHF) | 588(DF) | 23 | 13 |
| Thein et al.  (2014) (8) | Singaporean | case-control | ELISA | | Report review | 135 | | 27 | 108 | 4 | 3 |
| Karunakaran et al.  (2014) (9) | Kerala, India | case-control | ELISA | | Report review | 50 | | 10 | 40 | 4 | 1 |
| Wei et al.  (2016) (10) | Taiwan | case-control | RT-PCR+ ELISA | | Report review | 136 | | 20(Death) | 116(DHF) | 13 | 63 |
| Han et al.  (2017) (11) | Kuala Lumpur, Malaysia | case-control | ELISA | | Report review | 365 | | 22 | 343 | 3 | 9 |
| Kuo et al.  (2017) (12) | Gaoxiong, Taiwan | case-control | ELISA | | Report review | 669 | | 27(Death = 2) | 642(No death) | 9 | 86 |
| Agrawal et al.  (2018) (13) | Hyderabad, India | case-control | ELISA | | On-site testing | 334 | | 117(Death = 5) | 217( No death) | 45 | 37 |
| Salim et al.  (2012) (14) | Singaporean | case-control | RT-PCR+ ELISA | | Report review | 2285 | | 818(DHF) | 263(DF) | 45 | 47 |
| Mirza et al.  (2016) (15) | Lahore, Pakistan | Retrospective | ELISA | | / | 563 | | 300(DHF) | 263(DF) | 141 | 105 |
| Lee et al.  (2006) (16) | Taiwan | case-control | RT-PCR+ ELISA | | Report review | 644 | | 232(DHF = 220, DSS = 12) | 412(DF) | 39(DSS = 5, DHF = 34) | 31 |
| Wei et al.  (2022) (17) | Malaysia | case-control | ELISA | | / | 468 | | 117 | 315 | 23 | 35 |
| Werneck et al.  (2018) (18) | Brazilian | retrospective cohort | ELISA | | / | 326380 | | 15993(DHF) | 310387(DF) | 4 | 57 |
| Lee et al.  (2020) (19) | Gaoxiong, Taiwan | case-control | ELISA | | Report review | 1533 | | 285(DSS = 11, DHF = 249) | 1248(DF = 506) | 93(DSS = 6, DHF = 70) | 199(DF = 70) |
| Bode et al.  (2006) (20) | Colorado, USA | Retrospective | RT-PCR+ ELISA | | Report review | 221 | | 168(WNM = 103, WNE = 65) | 53(WNF) | 36(WNM = 11, WNE = 25) | 5 |
| Staples et al.  (2012) (21) | USA | Retrospective | ELISA | | Report review | 1090 | | 708(WNND) | 382(WNF) | 217 | 31 |
| Racsa et al.  (2014) (22) | Texas, USA | Retrospective | ELISA | | / | 57 | | 32(WNND) | 25(WNF) | 8 | 3 |
| Baraniuk et al.  (2006) (23) | Houston, Texas, USA | nested case control | ELISA | | Report review | 189 | | 130(Death = 17, WNE = 113) | 59(WNF) | 45(Death = 9, WNE = 36) | 11 |
| Jean et al.  (2007) (24) | California, USA | Retrospective | ELISA | | Report review | 839 | | 305(WNND) | 534(WNF) | 60 | 39 |
| Snyder et al.  (2020) (25) | California, USA | Retrospective | RT-PCR+ ELISA | | Report review | 3109 | | 272(Death) | 2837(No death) | 950(Death) | 129(No death) |
| Khairallah et al.  (2007) (26) | Monastir, Tunisia | Retrospective | ELISA | | Report review | 38 | | 32(WNVR) | 6(WNF) | 22 | 1 |
| Vrioni et al.  (2014) (27) | Greece | case-control | ELISA | | / | 31 | | 25(WNND) | 6(WNF) | 7 | 0 |

ELISA (Enzyme-Linked Immunosorbent Assays): A qualitative and quantitative detection method for immune reactions by binding soluble antigens or antibodies to solid phase carriers and utilizing antigen antibody binding specificity; RT-PCR (Real-time polymerase chain reaction): A molecular biology technique for real-time amplification and detection of specific DNA or RNA sequences. WNM: West Nile meningitis; WNE: West Nile encephalitis; WNND: West Nile Neuroinvasive Disease; WNVR: West Nile virus-associated retinopathy.

**References**

1. Jisamerin J, Mohamedkalifa A, Gaur A, Geetha J, Sakthivadivel V. Dengue: A Neglected Disease of Concern. Cureus. 2021;13(10):e18500.

2. Chen CY, Lee MY, Lin KD, Hsu WH, Lee YJ, Hsiao PJ, et al. Diabetes mellitus increases severity of thrombocytopenia in dengue-infected patients. Int J Mol Sci. 2015;16(2):3820-30.

3. Boillat-Blanco N, Klaassen B, Mbarack Z, Samaka J, Mlaganile T, Masimba J, et al. Dengue fever in Dar es Salaam, Tanzania: clinical features and outcome in populations of black and non-black racial category. BMC Infect Dis. 2018;18(1):644.

4. Figueiredo MA, Rodrigues LC, Barreto ML, Lima JW, Costa MC, Morato V, et al. Allergies and diabetes as risk factors for dengue hemorrhagic fever: results of a case control study. PLoS Negl Trop Dis. 2010;4(6):e699.

5. Wang WH, Lin CY, Chang K, Urbina AN, Assavalapsakul W, Thitithanyanont A, et al. A clinical and epidemiological survey of the largest dengue outbreak in Southern Taiwan in 2015. Int J Infect Dis. 2019;88:88-99.

6. Abualamah WA, Banni HS, Almasmoum HA, Allohibi YA, Samarin HM, Bafail MA. Determining Risk Factors for Dengue Fever Severity in Jeddah City, a Case-Control Study (2017). Pol J Microbiol. 2020;69(3):331-7.

7. Mallhi TH, Khan AH, Adnan AS, Sarriff A, Khan YH, Jummaat F. Clinico-laboratory spectrum of dengue viral infection and risk factors associated with dengue hemorrhagic fever: a retrospective study. BMC Infect Dis. 2015;15:399.

8. Pang J, Thein TL, Leo YS, Lye DC. Early clinical and laboratory risk factors of intensive care unit requirement during 2004-2008 dengue epidemics in Singapore: a matched case-control study. BMC Infect Dis. 2014;14:649.

9. Karunakaran A, Ilyas WM, Sheen SF, Jose NK, Nujum ZT. Risk factors of mortality among dengue patients admitted to a tertiary care setting in Kerala, India. J Infect Public Health. 2014;7(2):114-20.

10. Wei HY, Shu PY, Hung MN. Characteristics and Risk Factors for Fatality in Patients with Dengue Hemorrhagic Fever, Taiwan, 2014. Am J Trop Med Hyg. 2016;95(2):322-7.

11. Md Sani SS, Han WH, Bujang MA, Ding HJ, Ng KL, Amir Shariffuddin MA. Evaluation of creatine kinase and liver enzymes in identification of severe dengue. BMC Infect Dis. 2017;17(1):505.

12. Kuo HJ, Lee IK, Liu JW. Analyses of clinical and laboratory characteristics of dengue adults at their hospital presentations based on the World Health Organization clinical-phase framework: Emphasizing risk of severe dengue in the elderly. J Microbiol Immunol Infect. 2018;51(6):740-8.

13. Agrawal VK, Prusty BSK, Reddy CS, Mohan Reddy GK, Agrawal RK, Sekher Srinivasarao Bandaru VC. Clinical profile and predictors of Severe Dengue disease: A study from South India. Caspian J Intern Med. 2018;9(4):334-40.

14. Pang J, Salim A, Lee VJ, Hibberd ML, Chia KS, Leo YS, et al. Diabetes with hypertension as risk factors for adult dengue hemorrhagic fever in a predominantly dengue serotype 2 epidemic: a case control study. PLoS Negl Trop Dis. 2012;6(5):e1641.

15. Baig Mirza AM, Fida M, Murtaza G, Niazi R, Hanif A, Irfan K, et al. Association of metabolic factors with dengue viral infection on admission triage which predict its clinical course during Lahore dengue epidemic. J Pak Med Assoc. 2016;66(9):1102-6.

16. Lee MS, Hwang KP, Chen TC, Lu PL, Chen TP. Clinical characteristics of dengue and dengue hemorrhagic fever in a medical center of southern Taiwan during the 2002 epidemic. J Microbiol Immunol Infect. 2006;39(2):121-9.

17. Ng WY, Atan R, Mohd Yunos N, Bin Md Kamal AH, Roslan MH, Quah KY, et al. A double whammy: The association between comorbidities and severe dengue among adult patients-A matched case-control study. PLoS One. 2022;17(9):e0273071.

18. Werneck GL, Macias AE, Mascarenas C, Coudeville L, Morley D, Recamier V, et al. Comorbidities increase in-hospital mortality in dengue patients in Brazil. Mem Inst Oswaldo Cruz. 2018;113(8):e180082.

19. Lee IK, Hsieh CJ, Lee CT, Liu JW. Diabetic patients suffering dengue are at risk for development of dengue shock syndrome/severe dengue: Emphasizing the impacts of co-existing comorbidity(ies) and glycemic control on dengue severity. J Microbiol Immunol Infect. 2020;53(1):69-78.

20. Bode AV, Sejvar JJ, Pape WJ, Campbell GL, Marfin AA. West Nile virus disease: a descriptive study of 228 patients hospitalized in a 4-county region of Colorado in 2003. Clin Infect Dis. 2006;42(9):1234-40.

21. Lindsey NP, Staples JE, Lehman JA, Fischer M. Medical risk factors for severe West Nile Virus disease, United States, 2008-2010. Am J Trop Med Hyg. 2012;87(1):179-84.

22. Racsa L, Gander R, Chung W, Southern P, Le J, Beal S, et al. Clinical features of West Nile virus epidemic in Dallas, Texas, 2012. Diagn Microbiol Infect Dis. 2014;78(2):132-6.

23. Murray K, Baraniuk S, Resnick M, Arafat R, Kilborn C, Cain K, et al. Risk factors for encephalitis and death from West Nile virus infection. Epidemiol Infect. 2006;134(6):1325-32.

24. Jean CM, Honarmand S, Louie JK, Glaser CA. Risk factors for West Nile virus neuroinvasive disease, California, 2005. Emerg Infect Dis. 2007;13(12):1918-20.

25. Snyder RE, Cooksey GS, Kramer V, Jain S, Vugia DJ. West Nile Virus-Associated Hospitalizations, California, 2004-2017. Clin Infect Dis. 2021;73(3):441-7.

26. Khairallah M, Yahia SB, Letaief M, Attia S, Kahloun R, Jelliti B, et al. A prospective evaluation of factors associated with chorioretinitis in patients with West Nile virus infection. Ocul Immunol Inflamm. 2007;15(6):435-9.

27. Vrioni G, Mavrouli M, Kapsimali V, Stavropoulou A, Detsis M, Danis K, et al. Laboratory and clinical characteristics of human West Nile virus infections during 2011 outbreak in southern Greece. Vector Borne Zoonotic Dis. 2014;14(1):52-8.
